# Astrocytic Ephrin-B1 Regulates Synapse Remodeling Following Traumatic Brain Injury

**DOI:** 10.1177/1759091416630220

**Published:** 2016-02-27

**Authors:** Angeliki M. Nikolakopoulou, Jordan Koeppen, Michael Garcia, Joshua Leish, Andre Obenaus, Iryna M. Ethell

**Affiliations:** 1 Biomedical Sciences Division, School of Medicine, University of California Riverside, CA, USA; 2 Cell, Molecular, and Developmental Biology graduate program, University of California Riverside, CA, USA; 3 Department of Pediatrics, School of Medicine, Loma Linda University, CA, USA

**Keywords:** astrocytes, hippocampus, ephrinB1, STAT3, synapse, traumatic brain injury

## Abstract

Traumatic brain injury (TBI) can result in tissue alterations distant from the site of the initial injury, which can trigger pathological changes within hippocampal circuits and are thought to contribute to long-term cognitive and neuropsychological impairments. However, our understanding of secondary injury mechanisms is limited. Astrocytes play an important role in brain repair after injury and astrocyte-mediated mechanisms that are implicated in synapse development are likely important in injury-induced synapse remodeling. Our studies suggest a new role of ephrin-B1, which is known to regulate synapse development in neurons, in astrocyte-mediated synapse remodeling following TBI. Indeed, we observed a transient upregulation of ephrin-B1 immunoreactivity in hippocampal astrocytes following moderate controlled cortical impact model of TBI. The upregulation of ephrin-B1 levels in hippocampal astrocytes coincided with a decline in the number of vGlut1-positive glutamatergic input to CA1 neurons at 3 days post injury even in the absence of hippocampal neuron loss. In contrast, tamoxifen-induced ablation of ephrin-B1 from adult astrocytes in ephrin-B1^loxP/y^ERT2-Cre*^GFAP^* mice accelerated the recovery of vGlut1-positive glutamatergic input to CA1 neurons after TBI. Finally, our studies suggest that astrocytic ephrin-B1 may play an active role in injury-induced synapse remodeling through the activation of STAT3-mediated signaling in astrocytes. TBI-induced upregulation of STAT3 phosphorylation within the hippocampus was suppressed by astrocyte-specific ablation of ephrin-B1 *in vivo*, whereas the activation of ephrin-B1 in astrocytes triggered an increase in STAT3 phosphorylation *in vitro*. Thus, regulation of ephrin-B1 signaling in astrocytes may provide new therapeutic opportunities to aid functional recovery after TBI.

## Introduction

Traumatic brain injury (TBI) occurs as a result of a closed or penetrating head injury by an external mechanical force, including a blast wave, impact, or penetration by a bullet (Maas et al., 2008). While advanced diagnostic and monitoring approaches have lead to a steady increase in the survival rate following brain injury, TBI triggers long-term neuropsychological changes and physical disabilities affecting nearly 1.5 million individuals in the United States each year. Considerable efforts have been devoted to developing treatments that enhance neuronal survival following brain injury. However, our understanding of the mechanisms that regulate injury-induced brain rewiring is limited. Brain injury can cause dramatic changes in brain synaptic connectivity that may promote functional recovery, but may also lead to cognitive and neuropsychological impairment (Langlois et al., 2004; Nortje and Menon, 2004). In addition to neuronal damage to the neocortex at the time of injury, other areas of the brain, such as the hippocampus, are susceptible to long-term anatomical and functional changes. Alterations in hippocampal circuits may contribute to memory loss and long-term behavioral changes following injury (Baldwin et al., 1997; Scheff et al., 2005; Yu and Morrison, 2010; Atkins, 2011).

Astrocytes can facilitate brain repair after injury by protecting neurons from glutamate excitotoxicity and regulating the blood-brain barrier; however, the role of astrocytes in rewiring neuronal networks is not well understood (Ito et al., 2006; Myer et al., 2006; Brown and Murphy, 2008; Benowitz and Carmichael, 2010; Mostany et al., 2010; Shields et al., 2011). Astrocytes undergo substantial changes in response to brain and spinal cord injury (Sofroniew, 2009), including cell hypertrophy (Wilhelmsson et al., 2006), enhanced proliferation and scar formation, increased expression of glial-fibrillary acidic protein (GFAP), and reexpression of the progenitor markers vimentin and nestin (Eddleston and Mucke, 1993; Pekny and Nilsson, 2005; Sofroniew, 2005). Although it is elusive whether these changes are beneficial or detrimental for brain recovery, STAT3 signaling has been implicated in reactive astrogliosis and astrocyte-specific STAT3 KO mice exhibited attenuated upregulation of GFAP, failure to exhibit astrocytic hypertrophy, and disruption of astroglial scar formation after spinal cord injury (Herrmann et al., 2008; O’Callaghan et al., 2014). STAT3 is also known to regulate astrocytic differentiation (Bonni et al., 1997), the formation of perineuronal astrocytic processes, and the expression of synaptogenic molecule TSP-1 (Tyzack et al., 2014).

EphB receptor tyrosine kinases and their ligands, ephrin-Bs, play an important role in neuronal connectivity and synaptogenesis (Ethell and Pasquale, 2005). More precisely, ephrin-B/EphB signaling pathways participate in cell–cell interaction and regulate a plethora of biological processes during development and in adulthood, such as axon guidance (Zimmer et al., 2003), synaptogenesis (Moeller et al., 2006; Segura et al., 2007), dendritic spine formation (Henkemeyer et al., 2003), and neurogenesis (Conover et al., 2000; Catchpole and Henkemeyer, 2011). Recent studies have linked several Ephs and ephrins to neurodevelopmental disorders, neurodegenerative diseases, and central nervous system injuries (Cisse et al., 2011; Georgakopoulos et al., 2011; Sanders et al., 2012; Coulthard et al., 2012; Overman et al., 2012; Van Hoecke et al., 2012; Ren et al., 2013; Barthet et al., 2013), but the mechanisms of ephrin-B/EphB signaling in neurologic diseases remain largely unexplored. Both ephrins and EphB receptors are shown to be upregulated in reactive astrocytes following injury (Sofroniew, 2009). An increase in ephrin-B1 expression has been reported in reactive astrocytes in the hippocampus after transection of entorhinal afferents (Wang et al., 2005). Similarly, ephrin-B2 and Eph receptor levels increase in astrocytes after spinal cord injury (Goldshmit et al., 2006) and the ablation of astrocytic ephrin-B2 leads to increased motor axon regeneration (Chen et al., 2013). Ephrin-B2 expression is also upregulated in microglia and astrocytes at the head of the optic nerve in glaucomatous DBA/2 J mice coinciding with the loss of retina ganglia cell axons (Du et al., 2007). The upregulation of ephrins in astrocytes most likely affects the Eph receptor activity in neurons. Indeed, increased EphB3 has been reported in regenerating axons after optic nerve injury and EphB3 loss impeded axonal regeneration (Liu et al., 2006). In stroke, ephrin-A5 upregulation in reactive astrocytes is associated with a significant increase in the phosphorylation of EphA receptors in peri-infarct tissue (Overman et al., 2012) and EphB1 levels are increased during axonal sprouting following stroke (Li and Carmichael, 2006). Although ephrin-A/EphA receptor interactions are implicated in astrocyte regulation of synaptic maintenance and remodeling after injury (Murai et al., 2003; Carmona et al., 2009; Filosa et al., 2009; Murai and Pasquale, 2011), the functional significance of astrocytic ephrin-B1 signaling in the regeneration of brain circuits after injury has not been investigated.

Here we report a transient upregulation of ephrin-B1 in the adult hippocampal astrocytes, but not microglia, following moderate TBI using a controlled cortical impact (CCI) model, concomitant with reactive astrogliosis. The upregulation of ephrin-B1 levels in reactive astrocytes in stratum radiatum (SR) area of CA1 hippocampus coincides with a decline in the number of vGlut1-positive glutamatergic input to CA1 neurons at 3 days post injury (dpi), followed by a significant downregulation of astrocytic ephrin-B1 at 7 dpi. Furthermore, targeted ablation of ephrin-B1 from adult astrocytes accelerates the recovery of vGlut1-positive glutamatergic input to CA1 neurons after TBI, suggesting that astrocytic ephrin-B1 may play an active role in injury-induced synapse remodeling. Finally, our studies suggest that astrocytic ephrin-B1 may act through the activation of STAT3-mediated signaling in astrocytes as the activation of ephrin-B1 in cultured astrocytes induces phosphorylation of STAT3, whereas astrocyte-specific deletion of ephrin-B1 suppresses STAT3 activation in the hippocampus following TBI *in vivo*.

## Materials and Methods

### Mice and Tamoxifen Injections

GFAP-ERT2^Cre/+^ (Jax#012849) male mice were crossed with ephrinB1^loxP/+^ female mice (exons 2 through 5 of *efnb1* gene are floxed by loxP sites, Jax#007664) to obtain GFAP-ERT2^Cre/+^ephrinB1^loxP/y^ (KO) or GFAP-ERT2^Cre/+^ (WT) male mice. WT and KO littermates male mice older than 8 weeks received tamoxifen intraperitoneally (1 mg; dissolved at 5 mg/ml in 1:9 ethanol/sunflower oil mixture) once a day for 7 consecutive days. Animals received surgery (see later) 1 to 2 weeks after the first tamoxifen injections as we observed a significant downregulation of ephrin-B1 expression in hippocampal astrocytes, but not neurons, of tamoxifen-injected GFAP-ERT2^Cre/+^ephrinB1^loxP/y^ during this period. We did not detect any changes in ephrin-B1 levels in astrocytes or neurons in GFAP-ERT2^Cre/+^ephrinB1^loxP/y^ noninjected or injected with sunflower oil without tamoxifen. Cre and ephrin-B1 immunoreactivities were analyzed in GFAP-ERT2^Cre/+^ephrinB1^loxP/y^ (KO) and GFAP-ERT2^Cre/+^ (WT) mice. GFAP-ERT2^Cre/+^ephrinB1^loxP/y^ animals showed a detectable Cre immunoreactivity and at least twofold reduction of ephrin-B1 immunoreactivity in astrocytes following tamoxifen injection. For expression of YFP in GFAP+ cells, Rosa-STOP_loxP_YFP animals were also crossed with GFAP-ERT2^Cre/+^ephrinB1^loxP/y^ or GFAP-ERT2^Cre/+^ mice. All genotypes were confirmed by polymerase chain reaction (PCR) analysis of genomic DNA isolated from mouse tails. Mice were maintained in an AAALAC accredited facility under 12-h light/dark cycles and fed standard mouse chow. All mouse studies were done according to NIH and Institutional Animal Care and Use Committee guidelines.

### Surgery

**Figure 1.**
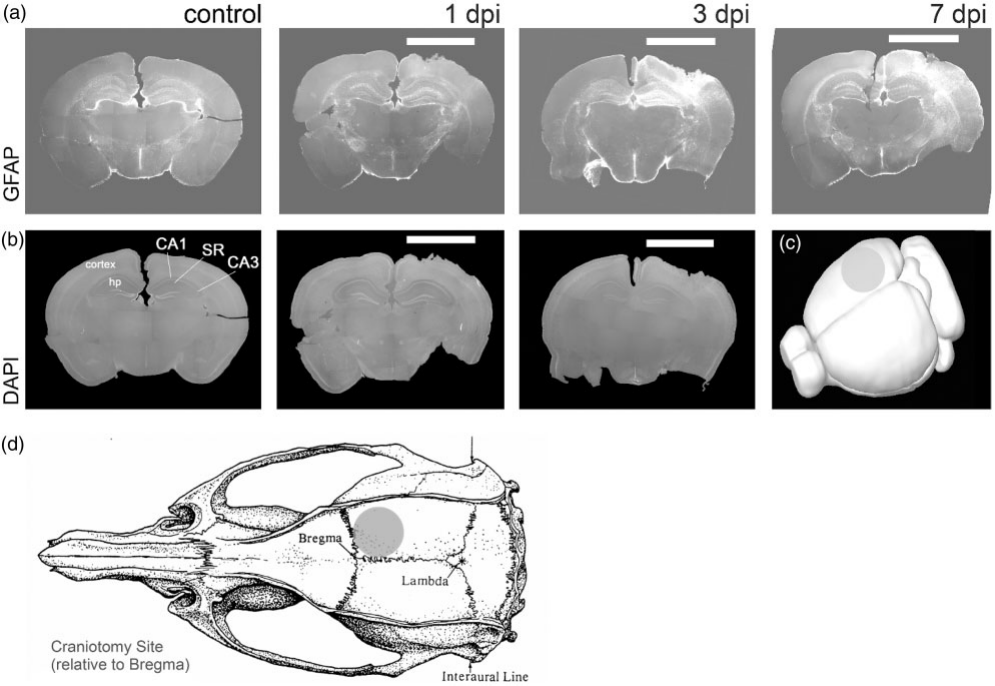
Reactive astrogliosis is observed in the hippocampus at 3 and 7 days following moderate CCI. Fluorescent images show GFAP immunoreactivity (a) and DAPI labeling (b) in coronal brain sections of control mice at 1, 3, or 7 dpi. The location of the impact area is indicated by a white bar. Note that reactive astrogliosis was observed in the ipsilateral cortex and hippocampus after moderate CCI, which leads to neuronal loss near cortical impact but not in the hippocampus. (c) 3D brain reconstruction shows the size and location of the impact. SR, stratum radiatum area of CA1 hippocampus; hp, hippocampus; CA1, CA1 layer; CA3, CA3 layer. (d) TBI location and size.

Two-month-old C57BL/6 wild-type, GFAP-ERT2^Cre/+^ephrinB1^loxP/y^ (KO) or GFAP-ERT2^Cre/+^ (WT) male mice received a unilateral CCI after a 4-mm craniotomy over the right parietal cortex (impactor center at Bregma: anterior-posterior, −1.50 mm, medial lateral 1.50 mm) using a stereotaxically positioned 3 mm diameter stainless steel tipped piston (Figure 1(d)). CCI (3 mm diameter tip, 1 mm depth, 6.0 m/s speed, 250 ms dwell) was then delivered to the cortical surface using an electromagnetically driven piston, resulting in cortical compression and some loss of cortical tissue. Moderate TBI in C57Bl6 mice results in a lesion volume of 2.51 ± 0.52% and 2.40 ± 0.46% (mean ± *SEM*; % of brain volume) at 3 and 7 days post injury (dpi), respectively, showing no hippocampal damage. Involvement of the hippocampus irrespective of the depth was considered severe TBI. Mice recovered quickly after TBI and demonstrated full activity within few hours after surgery, Sham mice received craniotomy without TBI. Mice with moderate CCI were analyzed in these studies. Animals were sacrificed at 1, 3, and 7 days post TBI or craniotomy. Untreated animals were sacrificed at similar timepoints as TBI animals for comparison.

### Immunohistochemistry

Animals were anesthetized with isoflurane and transcardially perfused first with 0.9% NaCl followed by with 4% paraformaldehyde in 0.1 M phosphate-buffered saline (PBS), pH = 7.4. Brains were postfixed overnight in 4% paraformaldehyde/0.1 M PBS and 100 -µm coronal brain sections were cut with a vibratome. We observed no neuronal loss or apoptotic nuclear morphology in the hippocampus of these animals at 1, 3, or 7 dpi by assessing nuclear morphology with 4'6-diamidino-2-phenylindole (DAPI) staining. To determine ephrin-B1 immunoreactivity in the hippocampus, double immunostaining was performed using goat antibodies against ephrin-B1 (1:50, BD Pharmingen). The specificity of the ephrin-B1 immunoreactivity in astrocytes was confirmed by the depletion of anti-ephrin-B1 antibody against ephrin-B1-Fc coupled to protein-A agarose (not shown). Astrocytes were labeled with conjugated Cy3-anti-GFAP (1:500, Sigma) and microglia with rabbit anti-Iba1 (1:1,000, Wako) antibodies to identify changes in glial phenotype. Cre expression was analyzed with mouse anti-Cre antibody (1:100, EMD Millipore). Presynaptic boutons were labeled by immunostaining for the excitatory synapse marker vesicular glutamate transporter 1 (vGLUT1) using rabbit anti-vGLUT1 (1µg/4μl; 482400; Invitrogen). Secondary antibodies used were Alexa Fluor 594-conjugated donkey anti-mouse IgG (4 µg/ml; Molecular Probes), Alexa Fluor 647-conjugated donkey anti-rabbit IgG (4 µg/ml; Molecular Probes), or Alexa Fluor 488-conjugated donkey anti-goat IgG (4 µg/ml; Molecular Probes). Sections were mounted on slides with Vectashield mounting medium containing DAPI (Vector Laboratories Inc.).

### Imaging

Confocal images from the ipsilateral and contralateral hemispheres were captured with a Leica SP2 confocal laser-scanning microscope using a series of high-resolution optical sections (1,024 × 1,024-pixel format) that were captured with a 20× (immunohistochemistry) or 63× (synaptogenesis) water-immersion objective (1.2 numerical aperture), 1 × zoom at 1 -µm step intervals (z-stack of 11 optical sections). All images were acquired under identical conditions. Each z-stack was collapsed into a single image by projection, converted to a tiff file, encoded for blind analysis, and analyzed using Image J Software. Three adjacent projections from SR were analyzed per brain slice from at least three animals/group. Cell area, integrated fluorescent intensity, and cell perimeter were determined for each GFAP-positive and ephrinB1-positive cells (100–300 astrocytes, 5–11 images, 3–4 mice per group). For the analysis of vGlut1 immunolabeling, at least six sequential images were captured for selected area at 1-µm step intervals, each image in the series was threshold-adjusted to identical levels and the puncta were measured using ImageJ. Three adjacent areas from SR were imaged and analyzed per brain slice from at least three animals/group.

### Cell Culture

Astrocytes were isolated from WT or ephrinB1^loxP^ mouse hippocampi at P0-P1 as previously described (Barker et al., 2008). Briefly, hippocampi were treated with 0.1%trypsin/ethylenediaminetetraacetic acid (EDTA) solution for 25 min at 37℃ and mechanically dissociated. Cells were plated on cell culture flasks and cultured in DMEM containing 10% fetal bovine serum (FBS) and 1% pen-strep, under 10% CO_2_ atmosphere at 37℃. To achieve purified astrocyte cultures (>95% astrocytes) cells were shaken after 4 days *in vitro* (DIV) for 1 h. After shaking, the media were removed and cells were washed twice with 0.1 M PBS (pH 7.4). Cells were then treated with 0.1% trypsin/EDTA solution for 20 min at 37℃ and plated on 10-cm Petri dishes with DMEM containing 10% FBS. Once confluent astrocytes were trypsinized and plated on six-well plates at a density of 1.2 × 10^6^ per plate and cultured for 2 days before being transfected with pEGFP, pEGFP and pcDNA-ephrinB1, or pEGFP and pcDNA-Cre plasmids using Lipofectamine according to the manufacturer’s instructions (Invitrogen, 11668-019). Astrocytes were treated with preclustered EphB2-Fc to activate ephrin-B1 or control Fc and processed for Western blot as described later.

### Ephrin-B1 Induction *In Vitro*

Preclustered EphB2-Fc or Fc were generated by incubating EphB2-Fc (R&D Systems) or Fc (R&D Systems) with goat anti-human IgG (Jackson ImmunoResearch) for 1 h at 4℃. Transfected astrocytes were stimulated with 2.5µg/ml EphB2-Fc or 2.5ug/ml Fc for 15 min. Cells were then lysed with lysis buffer: (in mM) 25 Tris-HCl, 150 mM NaCl, 5 EDTA, 1% Triton-X, 1 sodium pervanadate, and protease inhibitor mixture (1:100, Sigma, P8340).

### Western Blotting

The hippocampi were removed from each mouse (*n* = 4 mice per group), frozen, and stored at −80℃. Brain tissues were homogenized in cold lysis buffer: 50 mM Tris-HCl (pH = 7.4), 150 mM NaCl, 1 mM EDTA (pH = 8.0), 1% Triton X-100, 0.1% sodium dodecyl sulfate (SDS) containing protease inhibitor cocktail (1:100, Sigma, P8340), and 0.5 mM sodium pervanadate. The samples were rotated at 4℃ for at least 1 hr to allow for complete cell lysis and then cleared by centrifugation at 10,000 × g for 15 min at 4℃. Hippocampal and cell lysates were boiled in reducing sample buffer (Laemmli 2× concentrate, Sigma, S3401), and proteins separated on 8% to 16% Tris-glycine SDS-PAGE precast gels (Life Technologies, EC6045BOX). Proteins were transferred onto Protran BA 85 Nitrocellulose membrane (GE Healthcare) and blocked for 1 hr at room temperature in 5% skim milk (BioRad, #170-6404). Primary antibody incubations were done overnight at 4℃ with antibodies diluted in tris-buffered saline (TBS)/0.1% Tween-20/5% bovine serum albumin (BSA). Primary antibodies used were anti-STAT3 (1:2,000; Cell Signaling; 4904S) and anti-p-STAT3 (1:2,000; Cell Signaling; 9145P). Blots were washed 3 × 10 min with TBS/0.1% tween-20 and incubated with the appropriate horseradish peroxidase (HRP)-conjugated secondary antibodies for an hour at room temperature in a TBS/0.1% tween-20/5% BSA solution. The secondary antibodies used were α-rabbit-HRP at 1:5,000 and α-mouse-HRP at 1:5,000 (GE Healthcare). After secondary antibody incubations, blots were washed 3 × 10 min in TBS/0.1% tween-20 and developed with ECL Detection reagent (Thermo Scientific, #80196). For reprobing, membrane blots were washed in stripping buffer (2% SDS, 100 mM β-mercaptoethanol, 50 mM Tris-HCl, pH = 6.8) for 30 min at 56℃, then rinsed repeatedly with TBS/0.1% tween-20, finally blocked with 5% skim milk, and then reprobed. Developed films were then scanned and protein levels quantified by comparing band density values obtained using ImageJ. Two samples per group were run per blot and pSTAT3/STAT3 ratio was calculated for EphB2-Fc-treated samples and normalized to pSTAT3/STAT3 ratio of Fc-treated samples. Statistical analysis was performed using two-way analysis of variance (ANOVA) followed by post hoc pair-by-pair comparisons with Fisher’s Least Significant Difference (LSD) method.

### Quantitative Real-Time PCR

RNA was isolated from mouse hippocampi using Trizol reagent (Invitrogen), precipitated in isopropanol, and RNA concentration (ng/µl) was identified from the absorbance at 260 and 280 nm, detected by NanoDrop ND-1000 spectrophotometer (NanoDrop Technologies). cDNA was transcribed using the Reverse Transcription System (Promega) according to manufacturer’s instructions. To examine mRNA expression of ephrinB1 (F: ACCCTAAGTTCCTAAGTGGGA, R: CTTGTAGTACTCGTAGGGC), EphB2 (F: TACATCCCCCATCAGGGTGG, R: GCCGGATGAATTTGGTCCGC, GFAP (F: GCCACCAGTAACATGCAAGA, R: GCTCTAGGGACTCGTTCGTG), and vimentin (F: ATGCTTCTCTGGCACGTCTT, R: AGCCACGCTTTCATACTGCT), specific forward and reverse primers were used. Real-time PCR was carried out on an iCycler (Bio-Rad laboratories). Each reaction mixture contained 1 × Power SYBR Green PCR Master Mix (Life Technologies), and all the reactions were run in triplicate. The PCR amplification protocol was as follows: initial DNA Polymerase activation at 95℃ for 10 min, followed by 40 cycles with denaturation at 95℃ for 15 s, and annealing + extension at 60℃ for 1 min. Amplification was performed in a StepOne Real Time PCR System (96-well format) (Life Technologies) and analyzed by normalizing the expression of each gene to GAPDH within each tissue sample.

### Statistical Analysis

Both for *in vivo* and *in vitro* studies, the groups were compared using one-way or two-way ANOVA with Tukey’s or Bonferroni post hoc analysis, respectively, or paired Student’s *t*-test, and *p* values < .05 were taken as statistically significant.

## Results

The aim of this study was to determine whether ephrin-B1 levels are regulated in hippocampal astrocytes following moderate CCI and to investigate the role of astrocytic ephrin-B1 in synapse recovery after CCI *in vivo* using conditional ephrin-B1 KO mice.

### Moderate CCI Triggers a Transient Upregulation of Ephrin-B1 Immunoreactivity in Reactive Astrocytes in the Hippocampus

**Figure 2.**
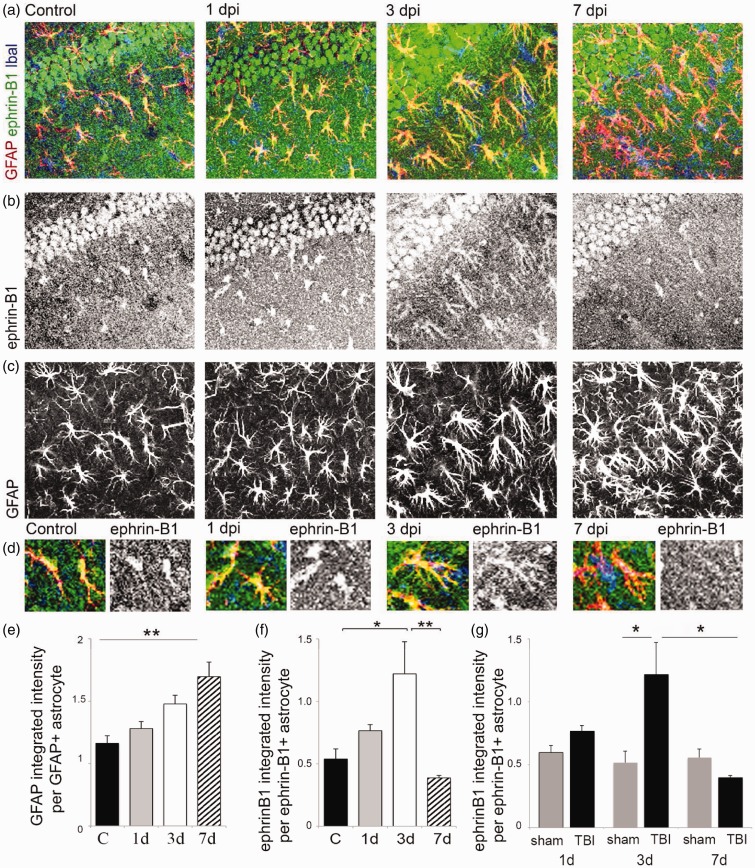
Ephrin-B1 immunoreactivity was significantly upregulated in reactive astrocytes in the hippocampus following moderate CCI. (a–d) Fluorescent images show GFAP-positive astrocytes (GFAP, red in a and d, gray in c), Iba1-positive microglia (Iba1, blue in a), and ephrin-B1 immunoreactivity (ephrin-B1, green in a, gray in b) in the SR area of the CA1 hippocampus in control, 1, 3, and 7 dpi. (d) High magnification images show examples of ephrin-B1-positive astrocytes. Note that ephrin-B1-positive immunoreactivity is found in the dendrites of CA1 neurons and astrocytes in SR area of CA1 hippocampus. (e–g) Graphs show GFAP immunoreactivity per GFAP-positive astrocyte (e) or ephrin-B1 immunoreactivity per ephrin-B1-positive astrocyte (f) in control (*n* = 682 cells, 9 images, 3 mice), 1 dpi (*n* = 385 cells, 5 images, 3 mice), 3 dpi (*n* = 732 cells, 11 images, 4 mice), and 7 dpi (*n* = 1217 cells, 9 images, 3 mice) or post-sham (*n* = 300–500 cells, 4–6 images, 3 mice). Note that ephrin-B1 immunoreactivity was not detected in all GFAP-positive cells. Error bars indicate *SEM*. Statistical analysis was performed using one-way ANOVA followed by Tukey’s post hoc analysis (e,f, *n* = 3–4 mice, *p < .05, **p < .01) or two-way ANOVA, followed by Bonferroni post hoc analysis (g, *n* = 3–4 mice, *p < .05).

**Figure 3.**
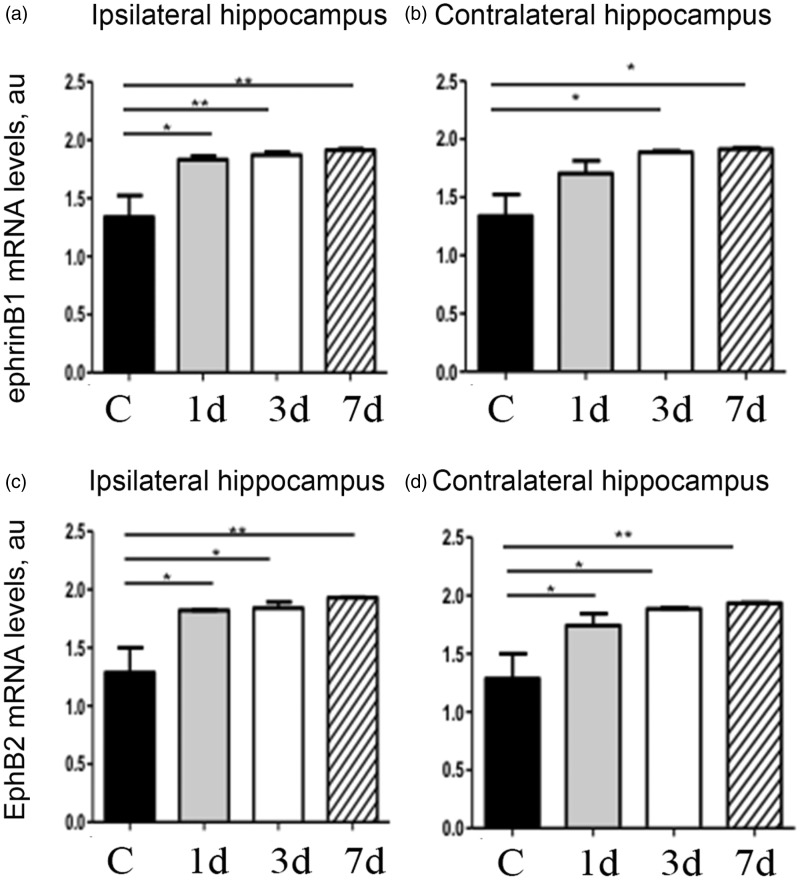
Moderate CCI causes an upregulation in gene expression of ephrinB1 and its receptor, EphB2 in the hippocampus. qPCR data from the hippocampi of control and TBI animals show that TBI causes an increase in ephrinB1 at all timepoints postinjury in the ipsilateral hemisphere; however, in the contralateral hemisphere, this increase is noticeable at 3 dpi. Similarly, we observe a concomitant increase in EphB2 levels at 1, 3, and 7 dpi both in the ipsi- and contralateral hemispheres. Graphs show mean ± *SEM* (*n* = 3, two-way ANOVA, *p < .05, **p < .01).

To induce TBI, mice received moderate unilateral CCI after craniotomy over the right parietal cortex (Figures 1(a) to (d)). As anticipated, we observed an increase in GFAP-immunoreactivity suggesting reactive astrogliosis at the site of injury (Figure 1(a)). Although we observed no neuronal loss or apoptotic nuclear morphology in the hippocampus of these animals at 1, 3, or 7 dpi by assessing nuclear morphology with DAPI staining (not shown), an increase in GFAP immunoreactivity was noted in the ipsilateral hippocampus at 3 dpi, with a significant upregulation of GFAP immunoreactivity at 7 dpi (Figures 1(a) and 2(c) and (e)). To examine whether ephrin-B1 levels were also upregulated in the reactive astrocytes after TBI, we performed immunostaining against ephrin-B1 and analysis of GFAP and ephrin-B1 immunoreactivity per area of astrocyte (Figure 2(a) to (f)). Our results show a significant increase of ephrin-B1 immunoreactivity in astrocytes at 3 dpi in the SR area of CA1 hippocampus as compared with controls (*p* < .05; Figure 2(f)) but not in cortical astrocytes around the impact area (not shown). Surprisingly, at 7 dpi, ephrin-B1 immunoreactivity in hippocampal astrocytes had subsided to control levels and was significantly lower than at 3 dpi (*p* < .01; Figure 2(f)). Interestingly, although ephrin-B1 immunoreactivity was reduced in astrocytes at 7 dpi, astrocytes remained reactive and showed higher GFAP immunoreactivity as compared with control astrocytes (*p* < .01; Figure 2(e)). Ephrin-B1 immunoreactivity was also slightly upregulated in CA1 neurons, but we could not detect ephrin-B1 immunoreactivity in microglial cells (blue, Figure 2(a)). To further verify that the changes described earlier are induced as a consequence of the brain injury, we compared TBI animals to animals that had received craniotomy without the impact (sham). Our data clearly show that craniotomy alone does not promote upregulation of ephrin-B1 immunoreactivity at any timepoint (Figure 2(g)). In contrast, ephrin-B1 immunoreactivity was significantly higher in hippocampal astrocytes at 3 dpi as compared with 3 days sham animals (*p* < .01; Figure 1(g)). In addition to immunohistological studies, we examined gene expression levels of ephrin-B1 and its receptor EphB2 in control and TBI animals. Our results show an upregulation of both ephrin-B1 and EphB2 expression in the hippocampus after TBI compared with control animals (Figure 3). The changes in the levels of ephrin-B1 and EphB2 receptor may affect transynaptic ephrin-B/EphB signaling and synaptic rewiring following TBI.

### Ephrin-B1 Upregulation in Hippocampal Astrocytes Coincides With a Decline in vGlut1-Positive Glutamatergic Innervation of CA1 Hippocampal Neurons

**Figure 4.**
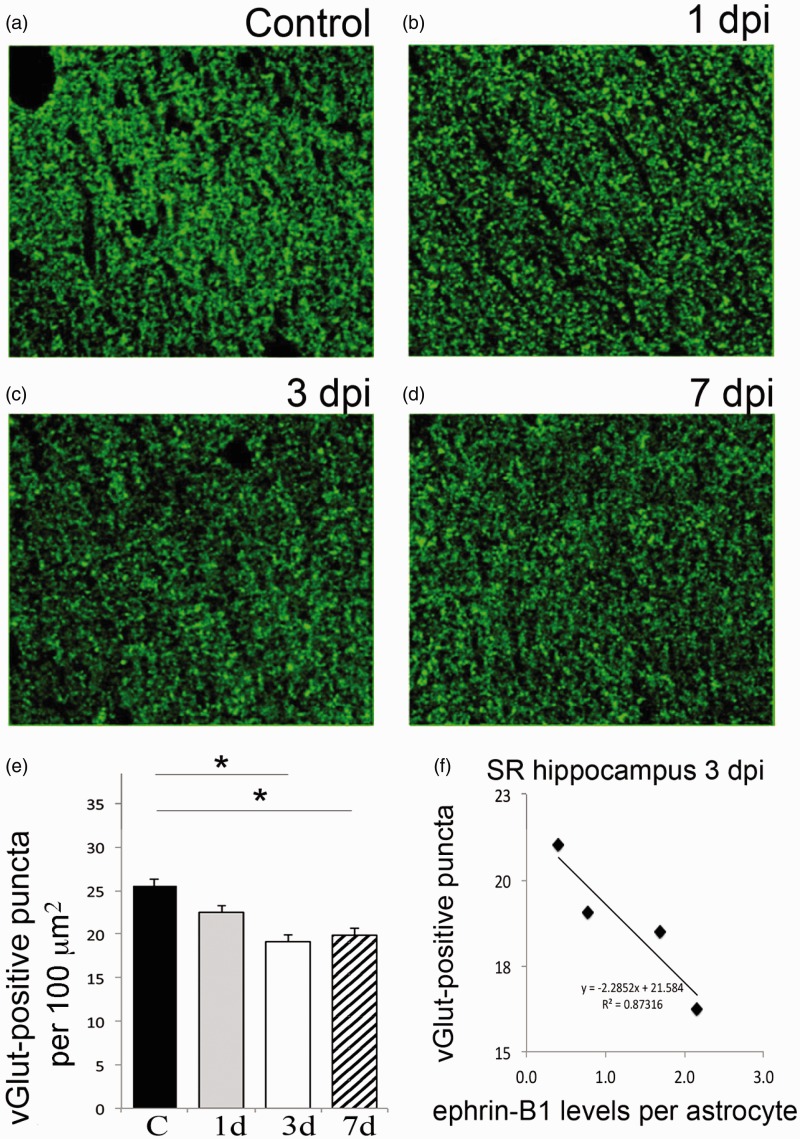
Moderate CCI triggers a significant downregulation of vGlut1-positive glutamatergic innervation of CA1 hippocampal neurons. We have analyzed vGlut1-positive puncta in SR area of the CA1 hippocampus, which immunolabels terminals of CA3 neurons innervating dendrites of CA1 neurons. (a–d) Fluorescent images show a reduced vGlut1-positive puncta in SR at 3 (c) and 7 dpi (d) as compared with control (a), suggesting a reduction in excitatory innervation of CA1 neurons following TBI. (e) Graph shows vGlut1-positive puncta per 100 µm^2^ area (mean ± *SEM*; *n* = 3–4 mice, one-way ANOVA followed by Tukey’s post hoc analysis, *p < .05). (f) Graph shows a negative correlation between the mean levels of ephrin-B1 per ephrin-B1-positive astrocyte (*x* axis, *n* = 100–300 astrocytes per animal) and the average number of vGlut1-positive puncta per 100 µm^2^ area (*y* axis, *n* = 50–100 images per animal) in SR area of CA1 hippocampus of four mice at 3 dpi. SR = stratum radiatum.

Although we observed reactive astogliosis, there was no significant neuronal loss noted in the hippocampus following CCI. Previously published results demonstrate that while most neurons are spared following moderate or mild CCI, the hippocampus undergoes substantial changes in synaptic organization (Scheff et al., 2005; Norris and Scheff, 2009; Gao et al., 2011). Therefore, we examined the changes in presynaptic Schaffer collateral input from CA3 to CA1 hippocampal neurons within the SR area of CA1 hippocampus following TBI by immunostaining against the excitatory presynaptic marker vGlut1 (Figure 4(a) to (d)). Our results show that at 3 dpi, when ephrin-B1 is significantly upregulated, the number of vGlut1-positive presynaptic boutons was significantly decreased in SR area of CA1 hippocampus (*p* < .05; Figure 4(e)). Furthermore, there was no further decline in the density of presynaptic boutons in the SR at 7 dpi, when ephrin-B1 levels were similar to controls (Figure 4(e)). Our results also show that there is a negative correlation between the levels of ephrin-B1 in astrocytes and the number of excitatory glutamatergic presynaptic input to CA1 neurons in SR at 3 dpi (Figure 4(f)), suggesting that astrocytic ephrin-B1 may play a role in synapse removal following TBI.

### Targeted Ablation of Ephrin-B1 From Astrocytes Promotes Fast Recovery of vGlut1-Positive Glutamatergic Innervation of CA1 Hippocampal Neurons Following CCI

**Figure 5.**
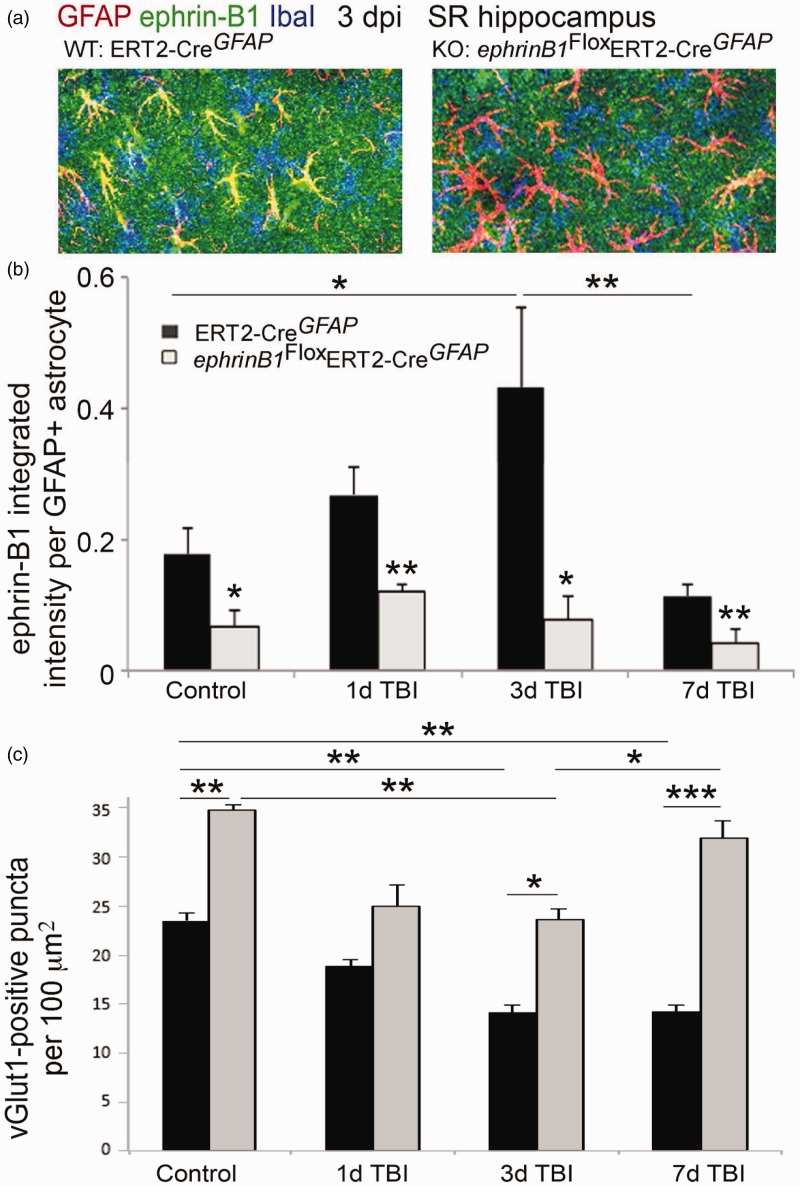
Astrocyte-specific deletion of ephrin-B1 triggers accelerated recovery of vGlut1-positive excitatory presynaptic sites in SR area of CA1 hippocampus at 7 dpi. (a) Confocal images show increased ephrin-B1 immunoreactivity (green) in WT (left panel), but not ephrin-B1 KO astrocytes (right panel) in the SR at 3 dpi. Note that the remaining ephrin-B1-positive immunoreactivity (green) in ephrin-B1 KO (right panel) represent ephrin-B1 expression in dendrites of CA1 neurons. (b, c) Graphs show integrated intensity of ephrin-B1 immunoreactivity per GFAP-positive astrocyte (b) and vGlut1-positive puncta per 100 µm^2^ area (mean ± *SEM*; *n* = 3–4 mice, two-way ANOVA, followed by Bonferroni post hoc analysis *p < .05, **p < .01, ***p < .001).

**Figure 6.**
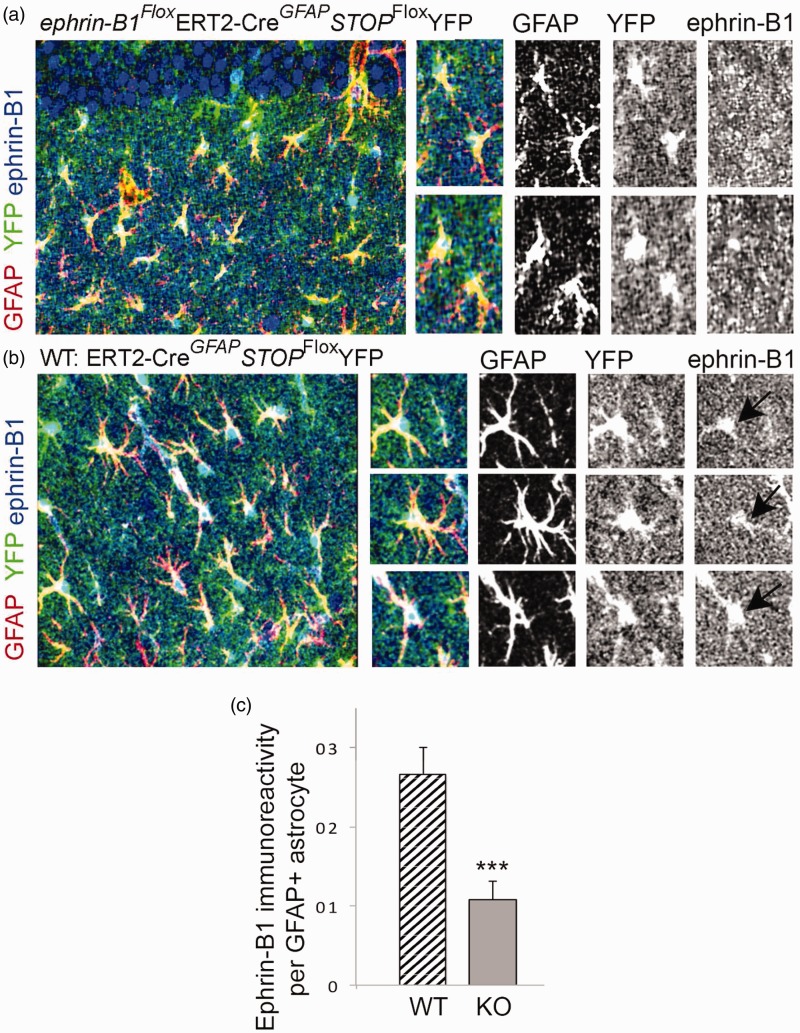
Specific ablation of ephrin-B1 from adult hippocampal astrocytes but not CA1 neurons *in vivo.* (a, b) Confocal images show YFP (green), GFAP (red), and ephrin-B1 (blue) in the SR area of CA1 hippocampus of *ephrin-B1*^Flox/y^GFAP-ERT2^Cre/+^*STOP*^Flox^YFP (KO, A) and control GFAP-ERT2^Cre/+^*STOP*^Flox^YFP (WT, B) mice. GFAP and ephrin-B1 levels were detected by immunostaining. YFP-positive WT astrocytes (black arrow, b) but not YFP-positive KO astrocytes (a) express ephrin-B1. Note that the ephrin-B1 deletion is specific to astrocytes and CA1 neurons express ephrin-B1 in both WT and KO mice. (c) Graph shows mean integrated intensity of ephrin-B1 immunoreactivity per GFAP-positive astrocyte in WT (*n* = 552 cells, 9 images, 3 mice) and KO (*n* = 520 cells, 9 images, 3 mice) groups (mean ± *SEM*; *n* = 3 mice). Statistical analysis was performed using paired Student’s *t* test (*p < .05, **p < .01).

To further establish a causal link between the upregulation in the levels of astrocytic ephrin-B1 and synapse reorganization after TBI, we developed a mouse model where ephrin-B1 was specifically ablated from adult astrocytes. A significant reduction in ephrin-B1 immunoreactivity was detected by immunostaining in GFAP-positive astrocytes of ephrinB1^loxP/y^GFAP-ERT2^Cre/+^ (KO) but not GFAP-ERT2^Cre/+^ (WT) mice treated with tamoxifen (Figure 5). The reduction in ephrin-B1 levels was also confirmed in adult astrocytes in Rosa-*STOP*^loxP^YFP/*ephrin-B1*^Flox/y^ERT2-Cre*^GFAP^* mice but not Rosa-*STOP*^loxP^YFP/ERT2-Cre*^GFAP^* mice following tamoxifen treatment (*p* < .001; Figure 6). Ephrin-B1 was still detected in the cell bodies and dendrites of CA1 neurons but not in YFP-positive astrocytes in SR area of CA1 hippocampus (Figure 6(a)). We found that astrocyte-specific deletion of ephrin-B1 triggered about three- to fivefold decrease in ephrin-B1 immunoreactivity in GFAP-positive astrocytes (Figures 5(b) and 6(c)).

Next, we examined whether the deletion of ephrin-B1 from astrocytes can prevent the loss of excitatory presynaptic innervation of CA1 neurons following TBI. TBI was performed 7 to 14 days after the first tamoxifen injection, at which point we see a selective ablation of ephrin-B1 from astrocytes (Figure 5(a)). Astrocytic ephrin-B1 levels were significantly reduced in SR area of CA1 hippocampus of ephrin-B1 KO as compared with WT mice at 1 dpi (*p* < .01), 3 dpi (*p* < .05), and 7 dpi (*p* < .01; Figure 5(a) and (b)). Although both WT and ephrin-B1 KO mice showed a significant loss of vGlut1-positive presynaptic innervation of CA1 neurons at 3 dpi as compared with control animals (*p* < .01), a significant recovery of vGlut1-positive presynaptic sites was observed in ephrin-B1 KO but not WT mice (Figure 5(c)). At 7 dpi, the number of vGlut1-positive presynaptic sites reached control levels in ephrin-B1 KO mice (*p* > .05), whereas the number of vGlut1-positive presynaptic sites remained low in WT mice (*p* < .001; Figure 5(c)). A higher number of vGlut1-positive presynaptic boutons was also seen at 3 dpi in ephrin-B1 KO mice as compared with WT mice (*p* < .05; Figure 5(c)). This difference was most likely a result of an overall increase in the number of vGlut1-positive presynaptic sites in ephrin-B1 KO mice as compared with WT mice prior to TBI (*p* < .01). Our results show that while a selective ablation of ephrin-B1 from astrocytes did not prevent the initial loss of vGlut1-positive presynaptic innervation of CA1 neurons in SR following CCI, it accelerated recovery of vGlut1-positive excitatory input to CA1 neurons at 7 dpi, suggesting negative effects of astrocytic ephrin-B1 on new synapse formation in CA1 hippocampus following TBI.

### Ephrin-B1 Regulates STAT3 Phosphorylation in Astrocytes Following TBI

**Figure 7.**
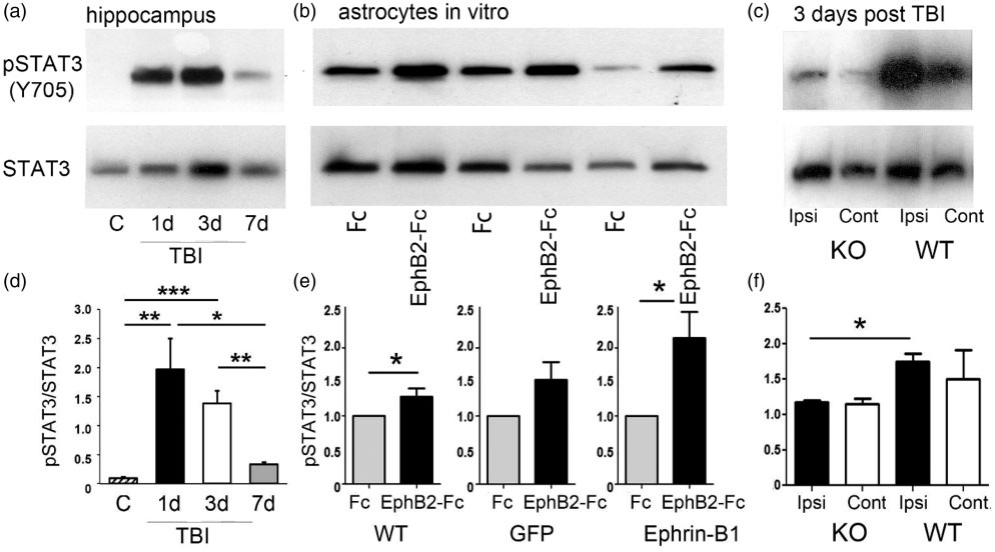
STAT3 phosphorylation in astrocytes is regulated by ephrin-B1. (a) Western blot analysis of pSTAT3 and STAT3 in the hippocampus of control, 1, 3, or 7 dpi. (b) Western blot analysis of pSTAT3 and STAT3 in the primary cultures of untransfected astrocytes (WT) and astrocytes overexpressing GFP (GFP) or ephrin-B1 (ephrin-B1) following the treatment with control Fc or EphB2-Fc for 15 min. (c) Western blot analysis of pSTAT3 and STAT3 in the ipsilateral (Ipsi) or contralateral (Cont) hippocampus of ephrin-B1 KO or WT at 3 dpi. The blots were first probed against pSTAT3 and then reprobed for total STAT3. (d–f) Graphs show pSTAT3/STAT3 (mean ± *SEM*; *n* = 3–4 cultures for E, Student’s *t* test, *p < .05; *n* = 3–4 mice for d and f, one-way ANOVA followed by Tukey’s post hoc analysis, *p < .05, **p < .01, ***p < .001). The levels of pSTAT3 and total STAT3 in EphB2-Fc-treated cultures were normalized to Fc-treated cultures (e).

STAT3 signaling plays an important role in reactive astrocytosis and may regulate protein expression in astrocytes following injury (Herrmann et al., 2008). Similar to previous work, our studies demonstrate an increase in the levels of phosphorylated STAT3 (pSTAT3) following brain injury (Figure 7(a)). Interestingly, the changes in pSTAT3 levels also coincided with increased ephrin-B1 immunoreactivity levels in astrocytes, showing increased pSTAT3 at 1 and 3 dpi as compared with control (*p* < .01 and *p* < .001), followed by its downregulation at 7 dpi (*p* < .01; Figure 7(d)). To establish a causal link between an upregulation in the levels of astrocytic ephrin-B1 and an increase in STAT3 phosphorylation, we examined the effects of ephrin-B1 activation in astrocytes on STAT3 phosphorylation *in vitro*. Our results show higher STAT3 phosphorylation (pSTAT3) in cultured astrocytes following ephrin-B activation with preclustered EphB2-Fc (*p* < .05; Figure 7(b) and (e)). We see a greater increase in pSTAT3 in astrocytes overexpressing ephrin-B1, suggesting that injury-induced increases in STAT3 phosphorylation may be mediated through the activation of ephrin-B1. Indeed, astrocyte-specific deletion of ephrin-B1 affected STAT3 phosphorylation following TBI. Our data show significantly lower levels of pSTAT3 in the ipsilateral hippocampus of ephrinB1-KO mice at 3 dpi when compared with WT animals (*p* < .05; Figure 7(c) and (f)). These studies suggest that ephrin-B1-dependent activation of STAT3 in astrocytes may contribute to astrocyte-mediated synapse remodeling following TBI.

## Discussion

**Figure 8.**
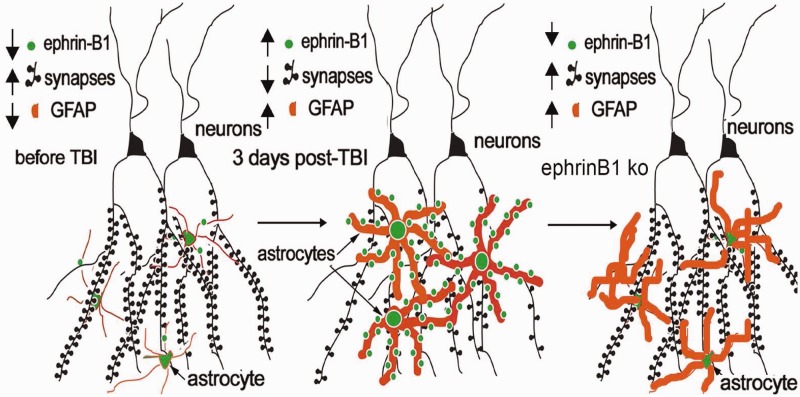
Visual depiction of hypothesis. Increased ephrin-B1 immunoreactivity in astrocytes coincide with a reduction in vGlut1-positive glutamatergic innervation of CA1 hippocampal neurons in SR at 3 dpi (middle panel), suggesting that astrocytic ephrin-B1 may regulate synapse reorganization in the hippocampus following CCI. Astrocyte-specific deletion of ephrin-B1 accelerates recovery of vGlut1-positive glutamatergic innervation of CA1 hippocampal neurons in SR after CCI (right panel).

The main findings of this study are as follows: (a) there is an increase in ephrin-B1 immunoreactivity in hippocampal astrocytes during synapse remodeling following moderate CCI in the absence of hippocampal neuron loss (middle panel, Figure 8), (b) specific deletion of ephrin-B1 from adult astrocytes accelerates the recovery of vGlut1-positive excitatory innervation of CA1 hippocampal neurons following injury (right panel, Figure 8), and (c) ephrin-B1 regulates STAT3 phosphorylation in hippocampal astrocytes and astrocyte-specific deletion of ephrin-B1 suppresses STAT3 activation in the hippocampus following CCI. Our results suggest the involvement of ephrin-B1 signaling in astrocyte-mediated remodeling of excitatory synapses following injury, which can be accomplished through ephrin-B1-mediated regulation of STAT3 signaling in astrocytes.

The CCI model of TBI is a well-established model that has been extensively used by many research groups to study both neuronal degeneration and plasticity following brain injury (Albensi et al., 2000; Immonen et al., 2009; Villapol et al., 2014). Numerous studies have documented the cellular and metabolic consequences of TBI (for a review, see Giza and Hovda, 2014). Quantitative magnetic resonance imaging has been used to assess the temporal evaluation of injury, where in the acute phase there is edema formation (Badaut et al., 2011) that peaks at 3 to 5 dpi. This edematous phase then resolves and results in increased astrogliosis as a consequence of ongoing neuronal cell death at the injury site. The predominant secondary injury mechanisms are believed to include increased [Ca^2+^]_i_, loss of metabolic regulation, and altered blood flow most prominently at the site of the injury (cortex in our model). However, here we studied the changes within the hippocampus away from the injury site in animals with moderate CCI when the hippocampus was not directly injured by the CCI. Although the roles of neurodegeneration and neuroplasticity are likely interwoven and are not mutually exclusive as the brain attempts to reestablish homeostasis, our moderate CCI model of TBI clearly results in cortical tissue loss independent of direct hippocampal tissue loss. Transient upregulation of ephrin-B1 immunoreactivity in hippocampal astrocytes, which is not observed in cortical astrocytes, is unlikely to be solely a direct response to the lesion, but rather a response within the hippocampus to deafferenation that occurs after cortical neuronal loss to establish homeostatic controls. Although astrocytic roles in water movement and glutamate recycling certainly can and likely do play a role in both neurodegeneration and plasticity, we propose that synaptic plasticity and reorganization are most likely mechanisms of astrocyte-derived ephrin-B1 reported here, considering its role in the normal synapse development.

Our studies suggest that astrocytic ephrin-B1 is a negative regulator of vGlut1-positive innervation in the CA1 hippocampus and ephrin-B1 expressing astrocytes may inhibit new synapse formation by competing with neuronal ephrin-B1. Our previous work demonstrated the importance of trans-synaptic neuronal ephrin-B/EphB interactions in the formation and maintenance of synapses *in vitro* and within the developing mouse brain (Henderson et al., 2001; Ethell et al., 2001; Takasu et al., 2002; Henkemeyer et al., 2003). In the adult brain, several EphB receptors, including EphB1, EphB2, and EphB3, are expressed at synapses (Torres et al., 1998; Buchert et al., 1999; Grunwald et al., 2001; Henderson et al., 2001). Inhibition of EphB receptor signaling by the expression of a kinase-inactive, dominant-negative form of EphB2 interferes with the maturation of postsynaptic dendritic spines in cultured hippocampal neurons (Ethell et al., 2001). Further supporting a physiological role of EphB receptors in synapse development, hippocampal neurons lacking multiple EphB receptors develop abnormal synapses *in vivo* (Henkemeyer et al., 2003). On the other hand, activation of EphB receptors in cultured hippocampal or cortical neurons with ephrin-B2 triggers the maturation of dendritic spines (Henkemeyer et al., 2003; Penzes et al., 2003) and induces clustering of EphB2 receptors together with NMDA receptors and other postsynaptic components (Dalva et al., 2000; Penzes et al., 2003). Although EphB2 contains a PDZ domain-binding site at its intracellular carboxy-terminal end, its association with the NR1 subunit of the NMDA receptor is direct and is mediated by the extracellular domain of EphB2 (Takasu et al., 2002; Nolt et al., 2011). The PDZ domain-binding motif of the EphB receptors may instead contribute to the localization of several PDZ domain-containing components to the postsynaptic density.

Postsynaptic proteins that interact with EphB receptors and possibly regulate their postsynaptic localization include the glutamate receptor-binding protein 1 (GRIP1), the protein interacting with C kinase 1 (Pick1), and the Ras-binding protein AF6 (Hock et al., 1998; Torres et al., 1998; Hoogenraad et al., 2005). Dalva et al. demonstrated that EphB2 also controls AMPA-type glutamate receptor localization through PDZ-binding domain interactions and also triggers presynaptic differentiation via its ephrin binding domain (Kayser et al., 2006). Thus, EphB receptors may promote the assembly of both pre- and postsynaptic proteins following their trans-synaptic interactions with ephrin-B ligand. Since both EphB receptors and ephrin-B ligands are membrane-bound, EphB/ephrin-B trans-interactions require cell–cell adhesion between cells expressing Eph receptors and those expressing ephrins. Interestingly, the EphB receptor may be either pre- or postsynaptic depending on the brain region (Contractor et al., 2002; Grunwald et al., 2004). EphB2 was found to be expressed in both CA1 and CA3 neurons and the disruption of interactions between presynaptic EphB2 on Schaffer collaterals and ephrin-B ligand on dendrites of CA1 hippocampal neurons was implicated in impaired hippocampal long-term potentiation observed in EphB2 KO mice (Grunwald et al., 2004). Therefore, it is possible that astrocytic ephrin-B1 detects presynaptic terminals that express the EphB2 receptor and prevent excessive innervation of CA1 hippocampal neurons by inhibiting growth of EphB2-containing sprouting fibers following injury. EphB receptor activation in axon fibers with ephrin-B ligand is well known to trigger the repulsion of ipsilateral retinal projections at the optic chiasm (Petros et al., 2009), to regulate growth of spinal motor axons (Wang and Anderson, 1997), and to induce collapse of axonal growth cones of embryonic hippocampal and cortical neurons *in vitro* (Lin et al., 2008; Srivastava et al., 2013). Ephrin-B/EphB receptor signaling has been also suggested to guide medial lateral motor column axons into the ventral limb (Luria et al., 2008) and to regulate the formation of intercortical connections through the guidance of axon fibers within the corpus callosum *in vivo* (Innocenti et al., 1995; Mendes et al., 2006). Indeed, we see an increase in the number of vGlut1-positive presynaptic fibers in SR area of CA1 hippocampus of astrocyte-specific ephrin-B1 KO mice as compared with WT mice following TBI, suggesting that astrocytic ephrin-B1 may prevent sprouting of CA3 axon fibers by repulsion.

It is also possible that ephrin-B1 expressing astrocytes directly target EphB-expressing presynaptic boutons. Ephrin-B-expressing astrocytes were shown to engulf neuronal EphB2 in mixed neuron/astrocyte cocultures at the sites of neuron-glial contacts (Lauterbach and Klein, 2006). However, functional significance of ephrin-B/EphB trans-endocytosis is not clear. Our studies are the first to show that specific deletion of ephrin-B1 from astrocytes accelerates v-Glut-positive glutamatergic innervation of CA1 hippocampal neurons, possibly due to reduced removal of vGlut1-positive terminals of sprouting fibers by astrocytes following TBI. Although the factors or molecular signals triggering astrocyte-mediated engulfment of synapses are not known, confocal microscopy revealed that both excitatory and inhibitory synapses are engulfed by astrocytes in the developing and adult mouse brain through phagocytic pathways (Chung et al., 2013). Other studies also reported a decrease in the number of synapses in CA1 hippocampus accompanied by decreased dendritic complexity at 3 dpi (Gao et al., 2011) and reduced excitability of CA1 hippocampal neurons at 7 dpi (Cohen et al., 2007). Thus, a vacant presynaptic EphB receptor from disassembled synaptic connections and a reduced synaptic activity may serve as an *eat me* signal to recruit ephrin-B1 expressing astrocytes and to initiate synapse engulfing process.

It is also possible that astrocytic ephrin-B1 may not be acting through neuronal EphB but activate EphB receptors in microglia to trigger microglia-mediated synaptic pruning. Microglia has been suggested to prune synaptic connections in the brain, and clathrin-coated spinules were observed at sites of contact between microglia and dendritic spines, axon terminals, or astrocytic processes (Tremblay et al., 2010; Schafer et al., 2012). The role of microglia in the developmental pruning of synapses is supported by studies showing reduced number of microglia and decreased synaptic pruning in the hippocampus of mice lacking the fractalkine receptor (Cx3cr1) (Paolicelli et al., 2011). However, mechanisms of glial-mediated synapse pruning are still not clear. Microglial activation was shown to be reduced following nerve injury in EphB1 KO mice (Cibert-Goton et al., 2013) and macrophages expressing EphB3 accumulated at the injury site after optic nerve crush injury (Liu et al., 2006). Future studies will establish the effects of astrocyte-specific overexpression of ephrin-B1 on microglial-dependent synapse pruning.

In addition to regulating EphB receptor *forward* signaling in neurons, ephrin-B1 is know to activate *reverse* signaling in ephrin-B-expressing cells through the activation of STAT3 (Bong et al., 2007). Therefore, it is possible that the effects of astrocytic ephrin-B1 on synapses are mediated through the activation of STAT3 signaling in astrocytes. Indeed, STAT3 is known to regulate astrocyte differentiation (Bonni et al., 1997), the formation of perineuronal astrocytic processes, and the expression of synaptogenic molecule TSP-1 (Tyzack et al., 2014). STAT3 signaling also plays an important role in reactive astrocytosis and may regulate protein expression in astrocytes following TBI (Herrmann et al., 2008). In fact, STAT3 phosphorylation is upregulated in astrocytes following TBI (Oliva et al., 2012) and astrocyte-specific deletion of STAT3 attenuated reactive astrogliosis and glial scar formation after injury (Wanner et al., 2013; O’Callaghan et al., 2014). Our results also show higher STAT3 phosphorylation (pSTAT3) in the hippocampus following TBI. Furthermore, we observed that astrocyte-specific ablation of ephrin-B1 suppressed injury-induced upregulation of STAT3 phosphorylation in the hippocampus *in vivo* and the activation of ephrin-B1 triggered an increase in STAT3 phosphorylation in cultured astrocytes.

Ephrin-B1-mediated activation of STAT3 may lead to synapse pruning through phagocytosis, as astrocyte-mediated pruning of synapses involves the activation of MEGF10 and MERTK phagocytic pathways (Chung et al., 2013), and STAT3 has been implicated in the complement-dependent phagocytosis (Pietrocola et al., 2013). Interestingly, the role of Rac-mediated pinocytosis in ephrin-B-dependent axon pruning was previously suggested via the activation of Grb4/DOCK180/Rac pathway (Marston et al., 2003; Parker et al., 2004; Xu and Henkemeyer, 2009; reviewed in Xu and Henkemeyer, 2012). The cytoplasmic tail of ephrin-B can become phosphorylated by the Src family of nonreceptor tyrosine kinases and recruits SH2/SH3 domain adaptor protein Grb4 following its interaction with EphB receptor. Grb4 adaptor protein links ephrin-B to Rac GTPase guanine exchange factor Dock 180. This pathway plays a critical role in ephrin-B3-mediated pruning of mossy fibers, axons of dentate granular cells (Xu and Henkemeyer, 2009), and EphB receptor-induced clustering and internalization of ephrin-B1 through a clathrin-mediated endocytotic pathway (Parker et al., 2004). As glial cells expressing ephrin-B are able to trans-endocytose full-length EphB2 receptor from the neighboring neurons in neuron/glial cocultures, it is possible that reactive astrocytes expressing ephrin-B1 are also involved in trans-endocytosis of EphB2 receptor containing presynaptic boutons following brain injury. Future studies will determine whether ephrin-B1 activation in astrocytes following its interaction with neuronal EphB receptor can induce engulfing of EphB/ephrin-B complex by astrocytes *in vivo* through Rac-mediated endocytosis.

However, the long-term effects of ephrin-B1 upregulation in astrocytes following TBI are still unclear. The reduction in vGlut1-positive innervation of CA1 neurons may contribute negatively to TBI recovery by eliminating existing connections that are necessary for appropriate hippocampal function. Conversely, elimination of synapses may improve recovery by reducing the amount of excitatory synapses and preventing glutamate excitotoxicity. In addition, accelerated restoration of excitatory synaptic innervation that we see in astrocyte-specific ephrin-B1 KO mice can lead to an early activation of cognitive and other processes during the window of vulnerability, which may actually be detrimental to long-term recovery (Griesbach et al., 2012; Shen et al., 2013). Long-term functional analysis will be preformed in future studies to determine if the short-term changes observed here lead to long-term benefits or deficits.

In summary, our studies show the role of astrocytic ephrin-B1 in the regulation of synapse remodeling in the hippocampus following brain injury. As the changes in synaptic circuits contribute to long-term neuropsychological changes and cognitive deficits observed in humans following brain injury, the regulation of ephrin-B1/EphB receptor signaling may provide new therapeutic opportunities to moderate synaptic connectivity and aid functional recovery after TBI.
